# Associations with perineal trauma during childbirth at home and in health facilities in indigenous municipalities in southern Mexico: a cross-sectional cluster survey

**DOI:** 10.1186/s12884-018-1836-8

**Published:** 2018-05-31

**Authors:** Abraham de Jesús-García, Sergio Paredes-Solís, Geovani Valtierra-Gil, Felipe Rene Serrano-de los Santos, Belén Madeline Sánchez-Gervacio, Robert J. Ledogar, Neil Andersson, Anne Cockcroft

**Affiliations:** 1 Centro de Investigación de Enfermedades Tropicales (CIET), Universidad Autónoma de Guerrero, Av. Pino s / n, Colonia El Roble, C.P.38640 Acapulco, Guerrero Mexico; 2 CIETinternational, 511 Avenue of the Americas #132, New York, USA; 3 Department of Family Medicine, McGill University, 5858 Chemin de la Côte-des-Neiges, Montreal, Canada

**Keywords:** Perineal tears, Episiotomy, Perineal trauma, Traditional midwife, Indigenous communities

## Abstract

**Background:**

Episiotomy and perineal tears remain common in vaginal deliveries. This study estimated the frequency of and factors associated with perineal tears, episiotomies, and postnatal infections among women in two predominantly indigenous municipalities in southern Mexico, where traditional midwives play an important role in women’s health.

**Methods:**

A cross-sectional study contacted women who gave birth in the previous three years. An administered questionnaire asked about place of delivery, birthing position, birth attendant, episiotomy, perineal tears, and wound infection after delivery. Cluster adjusted bivariate and then multivariate analysis examined factors potentially associated with self-reported perineal trauma (episiotomy and/or perineal tear). Key informant interviews sought insights into some of the findings.

**Results:**

Among women with a vaginal delivery, 71% (876/1238) of indigenous women and 18% (36/197) of non-indigenous women delivered at home. Some 17% (247/1416) of women overall, and 33% (171/525) of those delivering in a health facility, reported an episiotomy during delivery. Among 171 women reporting an episiotomy in a health facility, 30% (52) also reported a perineal tear. Overall, 13% (190/1412) of women reported they had a perineal tear during delivery, 17% (86/515) of those delivering in a health facility and 12% (104/897) of those delivering at home. A quarter of the women had self-reported perineal trauma during their last delivery, 38% (196/511) of those delivering in a health facility and 18% (160/893) of those delivering at home. In bivariate analysis, indigenous ethnicity, home delivery, upright posture in labour, and delivery by a traditional midwife were associated with a lower risk of perineal trauma, while primiparas had a higher risk. In the final multivariate model, delivery by a traditional midwife was protective (ORa 0.41, 95%CIca 0.32–0.54) and primiparity was a risk factor (ORa 2.01, 95%CIca 1.5–2.68) for perineal trauma. Women suggested that fear of bad treatment and being cut made them unwilling to deliver in health facilities.

**Conclusions:**

The rate of perineal trauma among women giving birth in indigenous communities could be reduced by efforts to decrease the use of episiotomies in health facilities, and by opening a dialogue with traditional midwives to increase their interaction with formal health services.

## Background

More than 30 years ago, the World Health Organisation recommended against routine episiotomy in uncomplicated vaginal deliveries [1]. Despite this, rates of episiotomy, especially among primiparous women, have remained very high in some regions and countries [2, 3], including countries in Latin America [4]. This is probably related to a continuing belief that episiotomy prevents severe perineal tears, especially among primiparas [5]. Analysis from a multi-country study in mainly low income countries estimated rates of severe (3rd and 4th degree) perineal tears of between 0.1 and 1.4%, with the rate in Mexico relatively high at 0.9% [6]. Reported risk factors for severe perineal tears include primiparity, high birthweight, instrumental delivery, and episiotomy [6, 7]. Both perineal tears and episiotomies can have important complications, including infection [8, 9], dyspareunia [10], urinary [11] and anal incontinence [12].

According to the National Population Council, in Mexico in 2014, 95% of deliveries were assisted by a doctor, 3% were assisted by a professional midwife or a traditional midwife, and 3% by a nurse or other person. In Guerrero, 84% of deliveries were assisted by a doctor, 9% by a professional midwife or a traditional midwife, and 4% by some other person. [13]. Most rural deliveries attended by traditional midwives take place at the woman’s house [14]. Other studies have reported that in Mexican states with a high proportion of indigenous populations like Chiapas and Oaxaca, more than half of all births are attended by traditional midwives [15, 16].

In Xochistlahuaca and Tlacoachistlahuaca municipalities of Guerrero state, 92 and 78% of the populations respectively are indigenous [17], mainly from the ethnic groups Na Savi (Mixteco) and Nancue Ñomndaa (Amuzgo). Only five of the 116 communities in Xochistlahuaca have health centres; a hospital in the municipal capital provides secondary care for the 28,000 residents. In Tlacoachistlahuaca, with 21,000 residents, seven of 53 communities have medical facilities.

Using baseline data from a study of neonatal survival, cultural safety and traditional midwifery, we analysed the frequency of perineal tearing, episiotomy, and perineal infection, and examined the factors associated with perineal trauma [18].

## Methods

A cross-sectional study included a random sample of 20 communities from a list of 169 communities in the two municipalities, stratified by ethnicity and access to health services, each with approximately 100 homes. Health promoters and local indigenous students who speak the indigenous language administered a questionnaire to all women who reported births within the last three years.

The questionnaire documented age at the time of the last delivery, education, language, parity, and socioeconomic status. It asked about where the delivery took place, who assisted the delivery, and the woman’s position during labour and delivery. Each woman reported whether she had a perineal tear or episiotomy during delivery, and if any perineal wound became infected, in response to the following questions: Did you have a tear in your vagina when you had your delivery? Did the doctor cut your vagina when you had your delivery? Was the wound infected – was there pus coming from the wound? We also asked women about the size of the baby, in terms of being bigger, the same size, or smaller than other babies in their community. We attempted to collect information about actual birth weight but more than half of the women did not know.

Double data entry with validation minimised keystroke errors. Analysis relied on CIETmap, a windows interface for the R programming language [19]. We defined “perineal trauma” as a self-reported episiotomy, a self-reported perineal tear, or both. We examined associations with the outcome of perineal trauma, in bivariate and then multivariate analysis, using the Mantel-Haenszel procedure [20]. Multivariate analysis began with a saturated model including the variables significantly associated with the outcome in bivariate analysis, removing the least significant associations one by one, until only variables associated with the outcome at the 95% confidence level remained. We report associations as odds ratios (OR) with 95% confidence intervals adjusted for the effect of clustering (95% CIca) using the Lamothe method [21].

After preliminary analysis of the survey findings, three of the researchers returned to the communities and carried out open interviews with key informants, to hear their views and experiences about some of the emerging issues. They interviewed two women who had recently given birth, two pregnant women, four traditional midwives (one of them a male), three husbands of women who had given birth or were pregnant, eight health workers from health centres, community basic hospitals and the regional general hospital, and five health service managers and planners. We audio-recorded the interviews. For the purpose of this analysis, we reviewed the responses relevant to perineal trauma and to women’s use of different types of services for pregnancy and delivery.

The study was approved by the Ethics Committee of Centro de Investigación de Enfermedades Tropicales (CIET), at the Universidad Autónoma de Guerrero in Mexico. Before each interview, the interviewer sought oral informed consent from the respondent. For respondents under the age of 16, their parents or legal guardians gave oral informed consent for them to participate.

## Results

We surveyed 1636 women, 720 in Xochistlahuaca and 916 in Tlacoachistlahuaca. Of these, 48% (783/1636) reported *Ñomndaa* as their mother tongue, 34% (563/1636) *Na savi,* and 18% (290/1636) Spanish; 47% (770/1636) of them said they could speak Spanish. More than one half of the women delivered at home (56%, 923/1636), 30% (493/1636) at the hospital, 8% (123/1636) in a health centre and 6% (90/1636) in a private clinic. Most (79%, 446/563) of *na savi* women and more than half (55%, 430/783) of *ñomndaa* women gave birth at home, compared with only 12% (36/290) of mestizo (non-indigenous) women (*p* < 0.0001). A quarter (26%, 181/706) of deliveries in health facilities were by caesarean section. These 181 deliveries are not included in the analysis of perineal trauma.

Table 1 shows the characteristics of the 1455 women with vaginal deliveries, in health facilities or at home. Overall, about two-thirds (923/1455) of these women delivered at home. Nearly three quarters (71%, 876/1238) of indigenous women delivered at home, while four out of five of the mestizo women (82%, 161/197) delivered in a health facility. One in five women was primiparous (21%, 310/1450) and 55% (172/310) of these delivered in a health facility, while only 31% (358/1140) of multiparous women delivered in a health facility. Almost all physician-assisted deliveries and three-quarters of nurse-assisted deliveries were in a health facility, while all deliveries assisted by a traditional midwife were at home. Women reported 151 home deliveries as being assisted by a relative; some of these relatives were also traditional midwives, but we are unable to say how many. The position for labour and delivery was reported by 1434 women. Most women delivering at home reported an upright position during labour, while nearly all who delivered in a health facility reported lying down during labour.

**Table 1:** Characteristics of 1455 women with vaginal deliveries in the last three years

Characteristics		Delivered in health facility (532)	Delivered at home (923)	All deliveries (1455)
		n (%)	n (%)	n (%)
Ethnic group	Aboriginal	362 (69)	876 (96)	1238 (86)
	Mestizo	161 (31)	36 (4)	197 (14)
Age at delivery	14 to 19 years old	66 (12)	111 (12)	177 (12)
	20 to 49 years old	466 (88)	811 (88)	1277 (88)
Able to read Spanish	Yes	365 (69)	330 (36)	695 (48)
	No	166 (31)	588 (64)	754 (52)
Civil status	Married/co-habiting	502 (95)	888 (97)	1390 (97)
	Single	26 (5)	24 (3)	50 (3)
Parity	Primipara	172 (32)	138 (15)	310 (21)
	Multipara	358 (68)	782 (85)	1140 (79)
Who attended delivery	Physician	460 (86)	20 (2)	480 (33)
	Nurse	59 (11)	17 (2)	76 (5)
	Health promotor	8 (2)	–	8 (0.5)
	Traditional midwife	–	643 (70)	643 (44)
	Relative	–	151 (16)	151 (10)
	Nobody	–	86 (10)	86 (6)
Position during labour and delivery	Upright/semi-upright	40 (8)	760 (83)	800 (56)
	Horizontal	482 (92)	152 (17)	634 (44)
Reported perineal tear	Yes	86 (17)	104 (12)	190 (14)
	No	429 (83)	793 (88)	1222 (86)
Reported episiotomy	Yes	171 (33)	76^a^ (9)	247 (17)
	No	354 (67)	815 (91)	1169 (83)
Reported perineal trauma (tear and/or episiotomy)	Yes	196 (38)	160 (18)	356 (25)
	No	315 (62)	733 (82)	1048 (75)
Reported infection of perineal wound	Yes	28 (15)	39 (24)	67 (19)
	No	165 (85)	121 (76)	286 (81)

^a^76 women who delivered at home reported having an episiotomy, although 59 of them were assisted in their delivery by a traditional midwife and only 7 by a doctor or nurse. It is extremely unlikely that the traditional midwife performed an episiotomy, so probably these women in fact had a perineal tear which they reported as an episiotomy (18 of them also reported a perineal tear)

### Perineal trauma

Overall, 17% (247/1416) of women reported they had an episiotomy during delivery (with or without a perineal tear), 33% (171/525) of those who delivered in a health facility and 9% (76/891) of those who delivered at home. It is likely that most of the 76 women who self-reported an episiotomy during home delivery had in fact experienced a perineal tear (Table 1).

Among those 171 women who delivered in a health facility and reported an episiotomy, 52 (30%) also reported having a perineal tear. Among health facility deliveries, a primipara was more likely to have an episiotomy than a multipara (OR 2.31, 95% CIca 1.53–3.50). Some 34% (117/343) of women who delivered in public hospitals reported an episiotomy, as did 24% (29/119) who delivered in health centres, and 42% (25/59) who delivered in private clinics.

Overall, 13% (190/1412) of women reported they had a perineal tear during their last delivery, 17% (86/515) of those who delivered in health facilities and 12% (104/897) of those who delivered at home (Table 1).

A quarter of the women had self-reported perineal trauma (episiotomy and/or perineal tear) during their last delivery, about one third of those who delivered in a health facility and one fifth of those who delivered at home (Table 1). Table 2 shows the results of bivariate analysis of factors potentially associated with reported perineal trauma. Aboriginal women, those who delivered at home, those who had an upright position during labour, and those assisted at delivery by a traditional midwife were significantly less likely to report perineal trauma. Primiparas were significantly more likely to report perineal trauma. Younger women were more likely to report perineal trauma, but this association was not statistically significant at the 5% level. Women reporting larger babies were not more likely to report perineal trauma.

**Table 2:** Bivariate associations with self-reported perineal trauma

Factor		With trauma		Without trauma		OR	95% CIca
		n	(%)	n	(%)		
Ethnicity	Aboriginal	255	(21)	942	(79)	**0.28**	**0.18–0.43**
	Mestizo	94	(49)	97	(51)		
Age at delivery	14–19 years old	57	(32)	120	(68)	1.48	0.96–2.3
	20–49 years old	310	(24)	967	(76)		
Place of delivery	Home	162	(18)	761	(82)	**0.34**	**0.21–0.56**
	Health facility	205	(39)	327	(61)		
Parity	Primiparae	120	(39)	190	(61)	**2.31**	**1.76–3.03**
	Multipara	245	(21)	895	(79)		
Position in labour	Upright	138	(17)	662	(83)	**0.38**	**0.24–0.61**
	Horizontal	224	(35)	410	(65)		
Size of baby	Bigger than average	96	(24)	302	(76)	0.92	0.70–1.19
	Average or smaller	251	(26)	723	(74)		
Birth attendant^a^	Traditional midwife	126	(19)	530	(81)	**0.38**	**0.22–0.65**
	Doctor/nurse	210	(39)	333	(61)		

Bold font indicates associations significant at the 5% level

*OR* Odds ratio, *95% CIca* Cluster adjusted 95% confidence intervals

^a^This excludes the few women who were assisted at delivery by a relative or by nobody

In the multivariate analysis, we included in the initial model all the variables shown in Table 2, except for ethnicity, which was strongly co-linear with place of delivery and birth attendant. Table 3 shows the final model from the multivariate analysis. Two variables remained independently associated with the outcome of self-reported perineal trauma. A woman whose delivery was assisted by a traditional midwife had less than half the risk of reporting perineal trauma compared with a woman whose delivery was assisted by a doctor or nurse. A primipara was twice as likely to report perineal trauma compared with a multipara.

**Table 3:** Final model of multivariate analysis of factors associated with self-reported perineal trauma (*n* = 1196)

Factor	ORna	ORa	95% CIca
Delivery assisted by traditional midwife	0.38	0.41	0.32–0.54
Primipara	2.31	2.01	1.5–2.68

*ORna* Unadjusted odds ratio, *ORa* Adjusted odds ratio, *95% CIca* Cluster adjusted 95% confidence intervals of ORa

### Infections of perineal wounds

Overall, 19% of women who reported perineal trauma (an episiotomy or a tear or both) reported that the wound became infected, as judged by the presence of pus coming from the wound (Table 1). Among women reporting a perineal wound, those who delivered at home were more likely to report that the wound became infected, compared with those who delivered in a health facility, but the difference was not significant at the 5% level (OR 1.90, 95% CIca 0.87–4.14). Considering all women with vaginal deliveries, 5% (44/878) who delivered at home reported an infected perineal wound, compared with 6% who delivered in a health facility (31/510); the difference was not significant at the 5% level (OR 0.80, 95% CIca 0.43–1.48). Given the small number of reported infections in our sample, we were not able to examine further potential associations with this outcome.

### Views of key informants

Some participants described bad experiences in health facilities.

“When I did not push hard enough, the staff told me they would put a condom into my vagina so that I would not get pregnant again. They told me to push and then they cut my part. I wish I had been accompanied (delivered) by the traditional midwife – then things would have been much better” (Woman who had recently delivered).

A patient explained that they don’t like to go to the hospital for delivery; they prefer to deliver at home assisted by the traditional midwife.

“In the health centre the delivery is rushed – it’s not the same as having the baby at home”.

Some women go to health facilities because of the monetary incentive (pregnant women get a monthly allowance that they can only claim if they are attending the health facility).

“If we don’t go, we don’t receive the money, that’s why we go to the hospital”.

Health workers considered that women should deliver in health facilities, but that ignorance sometimes prevented them doing so.

“We tell them that they should come here to deliver, it won’t cost them anything, and they will have a clean delivery, without complications.” (A staff physician).

“They don’t know, they are not used to getting care from health facilities” (A staff obstetrician).

Some health workers accepted that women might have other reasons for not attending health facilities, such as being scolded when they attended, or having embarrassing examinations.

“The women feel embarrassed by being examined by the doctor in the lithotomy position.” (A staff nurse).

An obstetrician described the steps he believed necessary to prevent infections during delivery, which might well be objectionable to women.

“The patient should come into the delivery room naked, and then we provide her with a hospital gown. Shaving the perineum is routine for all women delivering here”. (A staff obstetrician).

Another made clear that he believed episiotomy was necessary to avoid perineal tears.

“If you don’t do something, there can be a tear. We make an episiotomy to avoid tears.” (A staff obstetrician).

A health personnel expressed her belief that being indigenous is a risk factor for delivery complications.

“Yes, being indigenous is itself a risk factor for delivery complications, because of their culture, the myths and beliefs they have, and because of the difficulty of communication.” (A staff physician).

## Discussion

We found that women delivered by traditional midwives were less likely to have self-reported perineal trauma than those delivered by doctors or nurses, and this association remained when other potential risk factors for perineal trauma were taken into account. Women delivered at home by traditional midwives did not have the trauma of episiotomies, and they also had lower rates of perineal tears. Delivery in health facilities increased the risk of perineal trauma among the women in our study.

Our finding of less perineal trauma among women delivered by traditional midwives is compatible with other studies. In Yucatan, Mexico, 10% of women who delivered in hospital had perineal tears, compared with only 6% of those who delivered at home assisted by a traditional midwife [22]. Although in a very different context, a study from the United Kingdom found that women delivered by professional midwives working independently, were more likely to have an intact perineum than those delivered within the National Health Service system [23] and a study in Austria reported fewer episiotomies and less perineal trauma when care was led by professional midwives rather than by obstetricians [24].

In our study, although women who laboured and delivered in an upright or semi-upright position had lower rates of perineal trauma, this association did not remain in multivariate analysis. There is still debate about the benefits and risks of an upright posture during labour; a recent update of a Cochrane review of trials of different postures in second stage labour concluded that an upright posture in labour reduced episiotomies but possibly carried an increased risk of second degree perineal tears [25].

One third of the women in our study who delivered in a health facility reported an episiotomy. This episiotomy rate is lower than in countries of South East Asia, where a study found an overall rate of 65%, ranging from 47% in Malaysia to 91% in Thailand [3]. It is similar to a rate of 25% reported from a study of hospital discharge records in the USA [26]. Routine episiotomy is still practised in some countries in the persisting belief that it prevents severe perineal tears, especially among primiparas [5]. This is despite a body of evidence to the contrary. A systematic review of trials of selective versus routine episiotomy, recently updated, found less risk of severe perineal trauma when episiotomies were performed only when necessary, and concluded that routine episiotomy to prevent severe perineal tears is not justified and has no benefits for the mother or the baby [27]. One third of the women in our study who reported an episiotomy in a health facility also reported a perineal tear. A previous study of women delivering in Acapulco’s General Hospital found that 20% of women who reported having an episiotomy during vaginal delivery also reported a perineal tear [10].

In our study, primiparas were significantly more likely to have self-reported perineal trauma compared with multiparas and this association persisted in multivariate analysis. This was largely because primiparas delivering in a health facility were more than twice as likely to have an episiotomy compared with multiparas. Our key informant interviews with health workers suggested that this is because doctors perform episiotomies more routinely among primiparas, in the belief it will prevent perineal tearing.

Concerns about the way women are treated when they deliver in local health facilities, including high rates of episiotomy, might discourage women from delivering there. Key informants in our study communities described bad experiences in health care facilities, including one woman who described having her “part chopped” without warning. Interviews with health workers confirmed that they considered women should deliver in health facilities, but also exposed beliefs and practices that might well discourage women from doing so. Our study covered municipalities of predominantly indigenous populations with a particular world view regarding pregnancy, childbirth and postpartum, with a preference for home delivery assisted by a traditional midwife, similar to other indigenous groups in Mexico [14, 16].

In rural Mexico, traditional midwives play an important role in the care of pregnant women and can be an important link between the health care services and communities. The strong social and cultural identification between indigenous women and their traditional midwives is a good argument to consider incorporation of traditional midwives into health services in remote areas [28]. In our study sites, where just over half the deliveries took place at home assisted by a traditional midwife, key informant interviews indicated that traditional midwives encouraged pregnant women also to attend health facilities for antenatal care, and are interested in cooperating with official health services. For many years, researchers have called for the development of working linkages with traditional midwives to reduce the work load of government medical services while showing respect for indigenous cultures [29]. Our results suggest that increased cooperation with traditional midwives could reduce the rates of perineal trauma during delivery and the associated complications.

### Limitations

We relied on women’s self-reports of having an episiotomy or a perineal tear, for deliveries up to three years previously and did not have any means of verifying their reports. However, a study among women living in slum areas of Dhaka, Bangladesh, reported 82% agreement between the post-partum physical findings of physicians and maternal reporting of perineal tears [30]. As shown in Table 1, some women in our study who delivered at home assisted by a traditional midwife reported an episiotomy, when probably they experienced a perineal tear. Women judged that they had perineal tearing mainly because of the associated pain, as well as the subsequent presence of a wound, with or without infection, and because of what the person assisting the delivery told them. We have no reason to believe that traditional midwives were less likely than health workers in a facility to tell women if they had suffered perineal trauma. Women delivering in a health facility may have been less aware of tears because of the use of analgesia in this setting. We did not ask women about analgesia during their last delivery. Despite uncertainty about the frequency of perineal trauma based only on women’s self-reporting, we believe the analysis of the factors related to the trauma is valid.

We were not able to examine whether women with high birth weight babies were more likely to have perineal trauma because more than half the women did not know the birth weight of their baby; there was no association between self-reported large babies and perineal trauma. We did not ask women about the duration of labour, another potential risk factor for perineal trauma.

We defined infection of the perineal wound based on women’s self-report of pus coming from the wound. There was a higher rate of infections among home deliveries, although the difference was not statistically significant. The relatively small number of infections reported by the women in our sample meant we could not analyse the factors related to infections.

## Conclusions

Our results from the very periphery of the Mexican health system indicate that delivery assisted by traditional midwives carries a lower risk of perineal trauma compared with delivery assisted by a doctor or nurse in a health facility. The rate of perineal trauma among women giving birth in indigenous communities could be reduced by efforts to decrease the use of episiotomies in health facilities, and by opening a dialogue with traditional midwives to increase their interaction with formal health services.

## Abbreviations

95% CIca: Cluster adjusted 95% confidence interval
CIET: Centro de Investigación de Enfermedades Tropicales
OR: Odds ratio

## References

[1] World Health Organization (1985). Appropriate technology for birth. Lancet.

[2] Graham ID, Carroli G, Davies C, Medves JM (2005). Episiotomy rates around the world: an update. Birth.

[3] The SEA-ORCHID Study Group (2008). Use of evidence-based practices in pregnancy and childbirth: South East Asia Optimising reproductive and child health in developing countries project. PLoS One.

[4] Althabe F, Belizán JM, Bergel E (2002). Episiotomy rates in primiparous women in Latin America: hospital based descriptive study. BMJ.

[5] Trinh AT, Roberts CL, Ampt AJ (2015). Knowledge, attitude and experience of episiotomy use among obstetricians and midwives in Viet Nam. BMC Pregnancy Childbirth.

[6] Hirayama F, Koyanagi A, Mori R, Zhang J, Souza J, Gülmezoglu A (2012). Prevalence and risk factors for third- and fourth-degree perineal lacerations during vaginal delivery: a multi-country study. BJOG.

[7] Christianson LM, Bovbjerg VE, McDavitt EC, Hullfish KL (2003). Risk factors for perineal injury during delivery. Am J Obstet Gynecol.

[8] Johnson A, Thakar R, Sultan AH (2012). Obstetric perineal wound infection: is there underreporting?. Br J Nurs.

[9] Sule S, Shittu S (2003). Puerperal complications of episiotomies at Ahmadu Bello University teaching hospital, Zaria Nigeria. East Afr Med J.

[10] Solana-Arellano E, Villegas-Arrizón A, Legorreta-Soberanis J, Cárdenas-Turanzas M, Enzaldo de la Cruz J, Andersson N (2008). Dispareunia en mujeres después del parto: estudio de casos y controles en un hospital de Acapulco, México. Rev Panam Salud Publica.

[11] Rockner G (1990). Urinary incontinence after perineal trauma at childbirth. Scand J Caring Sci.

[12] Abbott D, Atere-Roberts N, Williams A, Oteng-Ntim E, Chappell LC (2010). Obstetric anal sphincter injury. BMJ.

[13] Encuesta Nacional de la Dinámica Demográfica 2014, Consejo Nacional de Población, Instituto Nacional de Estadística y Geografía. México. 2015. México, DF. Boletín de prensa núm. 271/15 available from: http://www.inegi.org.mx/saladeprensa/boletines/2015/especiales/especiales2015_07_1.pdf. Accessed 18 Dec 2017.

[14] Argüello-Avendaño HE, Mateo-González A. Parteras tradicionales y parto medicalizado, ¿un conflicto del pasado? Evolución del discurso de los organismos internacionales en los últimos veinte años. Revista LiminaR. Estudios Sociales y Humanísticos. 2014;12(2):13–29.

[15] Braine T (2008). Mexico’s midwives enter the mainstream. Bull World Health Organ.

[16] Sánchez Pérez HJ, Ochoa Díaz López H, Navarro i Giné A, Martín Mateo M (1998). La atención del parto en Chiapas, México: ¿dónde y quién los atiende. Salud Publica Mex.

[17] Tascon Mendoza J A, Solís Cervantes G R. Situación de salud de los pueblos indígenas y perspectivas de una atención intercultural. En Estado del desarrollo económico y social de los pueblos indígenas de Guerrero. Programa Universitario México Nación Multicultural. UNAM. México, D.F. 2009.

[18] Neonatal survival, cultural safety and traditional midwifery in indigenous communities of Guerrero State, Mexico. ISRCTN80090228 DOI 10.1186/ISRCTN80090228. Available at: http://www.controlled-trials.com/ISRCTN80090228?q=&filters=recruitmentCountry:Mexico,conditionCategory:Pregnancy%20and%20Childbirth&sort=relevance&offset=4&totalResults=4&page=1&pageSize=10&searchType=basic-search.

[19] Andersson N, Mitchell S (2006). Epidemiological geomatics in evaluation of mine risk education in Afghanistan: introducing population weighted raster maps. Int J Health Geogr.

[20] Mantel N, Haenszel W (1959). Statistical aspects of the analysis of data from retrospective studies of disease. J Natl Cancer Inst.

[21] Andersson N, Lamothe G (2011). Clustering and meso-level variables in cross-sectional surveys: an example of food aid during the Bosnian crisis. BMC Health Serv Res.

[22] Méndez González RM, Cervera Montejano MD (2002). Comparación de la atención del parto normal en los sistemas hospitalario y tradicional. Salud Publica Mex.

[23] Symon A, Winter C, Inkster M, Donnan PT (2009). Outcomes for births booked under an independent midwife and births in NHS maternity units: matched comparison study. BMJ.

[24] Bodner-Adler B, Kimberger O, Griebaum J, Husslein P, Bodner K (2017). A ten-year study of midwife-led care at an Austrian tertiary care center: a retrospective analysis with special consideration of perineal trauma. BMC Pregnancy Childbirth.

[25] Gupta JK, Sood A, Hofmeyr GJ, Vogel JP (2017). Position in the second stage of labour for women without epidural anaesthesia. Cochrane Database Syst Rev.

[26] Frankman EA, Wang L, Bunker CH, Lowder JL (2009). Episiotomy in the United States: has anything changed?. Am J Obstet Gynecol.

[27] Jiang H, Qian X, Carroli G, Garner P (2017). Selective versus routine use of episiotomy for vaginal birth. Cochrane Database Syst Rev.

[28] Cameron M, Andersson N, McDowell I, Ledogar RJ (2010). Culturally safe epidemiology: oxymoron or scientific imperative. Pimatisiwin.

[29] Castañeda Camey X (1992). Embarazo, parto y puerperio: conceptos y prácticas de las parteras en el estado de Morelos. Salud Publica Mex.

[30] Fronczak N, Antelman G, Moran AC, Caulfield LE, Baqui AH (2005). Delivery-related complications and early postpartum morbidity in Dhaka, Bangladesh. Int J Gynaecol Obstet.

